# Cystic Meningioma Simulating Arachnoid Cyst: Report of an Unusual Case

**DOI:** 10.1155/2014/371969

**Published:** 2014-06-26

**Authors:** Docampo Jorge, Gonzalez Nadia, Vazquez Claudio, Morales Carlos, Gonzalez-Toledo Eduardo

**Affiliations:** ^1^Fundación Científica del Sur, Hipólito Yrigoyen 8680, Lomas de Zamora, B1832BQS Buenos Aires, Argentina; ^2^Clínica IMA, Adrogue, B1846DSK Buenos Aires, Argentina; ^3^LSU School of Medicine, Shreveport, LA 71103, USA

## Abstract

The purpose of this paper is to show an unusual case of meningioma simulating arachnoid cyst on CT scan and MRI, diagnosed in a 63-year-old woman evaluated for headache and vision disorders. The meningioma shown is predominantly cystic with a small mural nodule enhancing after gadolinium and exhibiting diffusion restriction. Cystic portion of the tumor is hypodense on CT, and evidences fluid signal intensity on T1- and T2-weighted MR imaging.

## 1. Introduction

The objective of this paper is to show an unusual case of meningioma simulating an arachnoid cyst on CT scan and conventional MRI.

Meningiomas are the most common benign intracranial tumors, representing 13–18% of all intracranial neoplasms. They are more common in women (2-3 : 1), especially middle-aged women (40–60 years old) [1]. Generally, these are solid lesions, highly cellular and well vascularized.

## 2. Case Report

We present the case of a 63-year-old female patient, evaluated for intermittent headaches in the right temporoparietal region associated with blurred vision for the past two years, which had increased in the last month. As a relevant antecedent, she referred to a cranioencephalic trauma 9 years ago in a traffic accident, with impact on the right temporal lobe, with no associated lesions. At that time, she underwent a CT scan, reporting no intracranial hematic collections.

**Figure 1:**
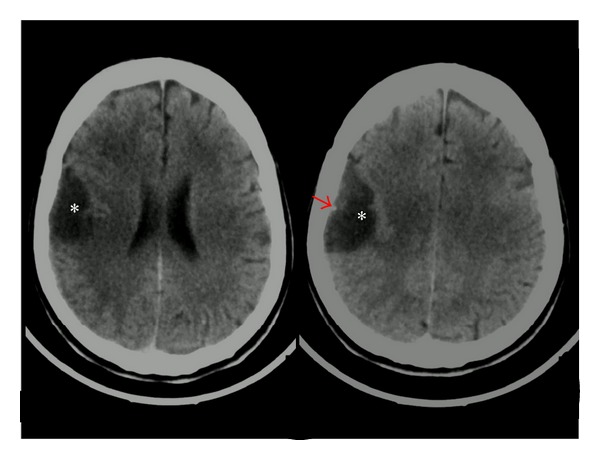
CT scan of the brain. A hypodense, extra-axial image is observed (asterisk), in the right frontoparietal region which imprints over the adjacent brain parenchyma. Lesion hypodensity is similar to the cerebrospinal fluid. A small mural hyperdense nodule (red arrow) is also identified.

Unenhanced CT scan was performed, identifying a hypodense image in the right frontoparietal region, with clear borders. A small mural hyperdense nodule was also identified (Figure 1). Such lesion was interpreted as an arachnoid cyst, due to its imagenologic features.

**Figure 2:**
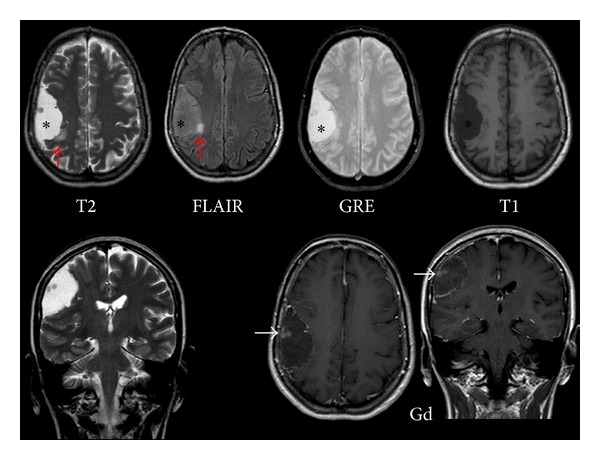
Gadolinium-enhanced brain MRI. The lesion is hypointense on T1- and hyperintense on T2-weighted images (asterisk). Signal intensity is high on FLAIR images probably due to the cyst protein content. A small mural nodule is observed. After gadolinium administration, enhancement of the small mural nodule (white arrow) and of the peripheral cystic area is observed. There is a small hyperintense area on T2-weighted and FLAIR images (red arrow) around the medial aspect of the cyst, probably related to the presence of reactive gliosis.

**Figure 3:**
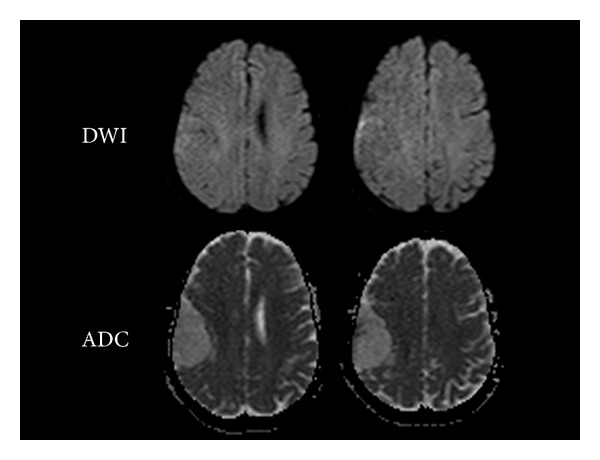
Diffusion-weighted image (DWI) and apparent diffusion coefficient (ADC) map image. The cystic portion of the lesion shows facilitated diffusion, although slightly more restricted than CSF.

Then, an MRI study was conducted using a high-field (1.5T) resonator, without and with gadolinium enhancement, applying diffusion technique and spectroscopy. Conventional sequences showed the lesion to be hypointense on T1-weighted sequences and hyperintense on T2-weighted and FLAIR sequences, with a dominant cystic component and a small mural nodule (Figure 2). After intravenous administration of contrast media, enhancement of the mural nodule and of the peripheral cystic area was observed. Diffusion showed impeded movement of the water molecules in the mural nodule and facilitated diffusion in the cystic area of the lesion (Figure 3). When compared to the cerebrospinal fluid (CSF), the cystic area showed less facilitated diffusion, probably due to its protein content.

**Figure 4:**
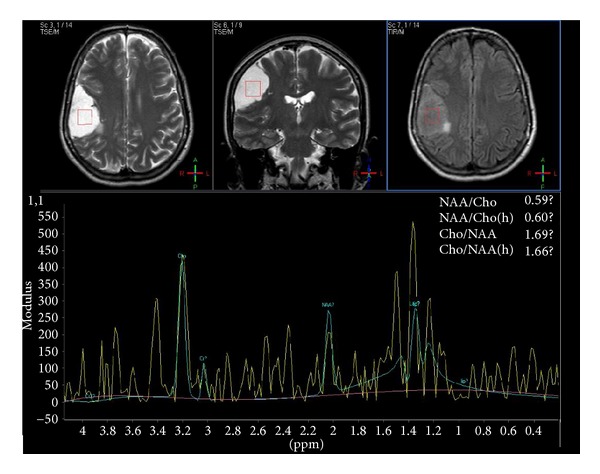
Spectroscopy. Increase in choline peak, decrease in NAA and creatine peaks, and increase in lactic and alanine peaks are observed.

MR spectroscopy showed an elevated choline peak and a decreased N-acetylaspartate peak at the mural nodule level. Furthermore, a double peak at 1.3 ppm and 1.5 ppm was observed, probably corresponding to an increase in the lactic and alanine peaks (Figure 4). With these results, glioma with cystic component, hemangioblastoma, and cystic meningioma were among the differential diagnoses.

**Figure 5:**
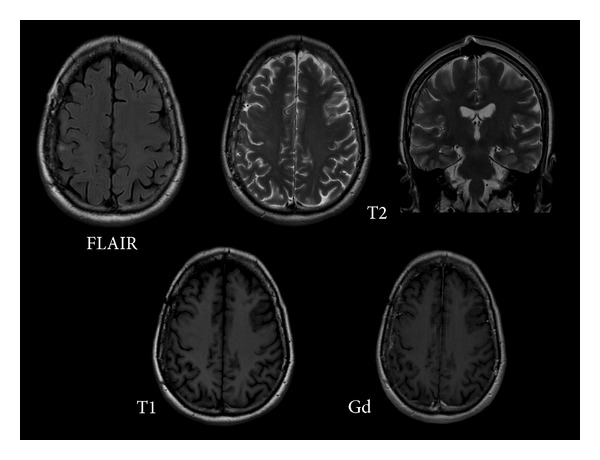
Control brain MRI. Three months after surgery a control brain MRI shows no lesion recurrence.

The patient underwent surgery, the lesion was completely resected, and macroscopic features were the presence of a cystic lesion containing a dense proteinaceous liquid, with a peripheral nodule of 8 mm in maximum diameter. Anatomic pathology revealed monomorphous cell proliferation, formed by medium sized cells with slightly hyperchromatic nuclei and moderate cytoplasm in a solid pattern, associated with the presence of numerous blood vessels with prominent walls. Immunohistochemistry revealed the following monoclonal antibodies: anti-EMA (+), Anti-Vimentin V9 (+), Anti-Cytokeratin Ae1/Ae3 (focal reactivity), and Anti-Ki-67 (+). Definitive diagnosis was cystic meningioma. Three months after surgery, brain MRI without and with gadolinium enhancement showed neither persistence nor meningioma recurrence (Figure 5).

## 3. Discussion

Meningiomas are usually benign tumors which originate from the meningothelial cells, extra-axial, frequently solid lesions, presenting typical imagenologic features both at CT scan and MRI in most cases (85%) [1]. Associated cysts are infrequent and generally confused with metastases or with high-grade glial neoplasms. Reported cases of cystic meningiomas in the literature show an incidence of approximately 2–4% and usually coincide with a cystic component associated with evident dural contact. The most common location is in the frontoparietal region [2]. Nauta described cystic meningiomas in 1979 and classified them into four types: Type I: central intratumoral cyst; Type II: peripheral intratumoral cyst; Type III: peritumoral cyst in the adjacent brain parenchyma; Type IV: cyst between the tumor and the adjacent brain parenchyma [3, 4]. The classification by Nauta et al. is considered to be the most useful [3, 5]. Our case would correspond to Type II.

Large eccentric cyst walls are associated with reactive gliosis or with collagen. Neoplastic cells are rarely found in the distal cyst wall; therefore, the entire cyst wall should be resected to prevent tumor recurrence [6–8].

Several authors have described the relation between meningioma and traumatic brain injury (TBI), especially Cushing, who cited 24 cases of a strong association between location of TBI and that of meningioma. In our case, the patient had a history of TBI with frontal and temporal lobe damage which coincided with the location of the meningioma, in keeping with Cushing's theory. However, the association between meningioma and TBI with frontal and temporal lobe damage as risk factor is currently controversial.

The literature describes some theories on the mechanism of cyst formation in this entity. Penfield believes that this is due to central degeneration within the tumor. Cushing suggested that cyst formation is due to the build-up of xanthochromic fluid at the periphery and that its coalescence leads to the formation of large cavities [3].

Intratumoral cysts may be the result of a degenerative process, ischemic necrosis, or hemorrhage [7]. They may also form as a result of active secretion from tumor cells [9], while other authors believe that cyst formation is due to the demyelination resulting from white matter edema and perfusion deficit [10].

Preoperative differentiation between cystic meningioma and other brain neoplasms such as gliomas, hemangioblastomas, and metastases with cystic component is difficult and frequently carried out after pathological examination [11, 12]. Brain angiography can help differentiate meningioma from other cystic lesions, since blood flow from the external carotid artery can be observed in cases of meningioma [13]. At MRI, meningiomas are often isointense on T1- and T2-weighted images and show homogeneous and intense contrast enhancement. The thickening of the adjacent dura (dural tail sign), when evident on MRI, and its extra-axial location, is highly useful for the preoperative diagnosis of cystic meningioma. However, cystic meningiomas can be difficult to differentiate from gliomas which partially show enhancement after the injection of contrast media or metastases, due to the presence of cyst which does not enhance and to focal edema. Contrast enhanced MRI can distinguish cystic walls infiltrated by tumor cells from those formed by gliotic tissue [5].

In diffusion techniques, meningiomas have different behavior according to the degree of cellularity, usually showing restriction to water molecules. In our case, the cystic component showed no restriction, presenting facilitated diffusion, but to a lesser extent than cerebrospinal fluid. The mural nodule showed restriction.

Spectroscopy reveals lower NAA, increased choline peak, and decrease in the NAA/Cho ratio. In addition, increase in lipid peaks and alanine can be observed at 1.5 ppm [14]. This was observed at spectroscopy in our case. Alanine might distinguish meningiomas from other neoplasms; however, it is not always present in all meningiomas [14].
