# Erratum to “Cystic Meningioma Simulating Arachnoid Cyst: Report of an Unusual Case”

**DOI:** 10.1155/2014/293805

**Published:** 2014-09-02

**Authors:** Jorge Docampo, Nadia Gonzalez, Claudio Vazquez, Carlos Morales, Eduardo Gonzalez-Toledo

**Affiliations:** ^1^Fundación Científica del Sur, Hipólito Yrigoyen 8680, Lomas de Zamora, B1832BQS Buenos Aires, Argentina; ^2^Clínica IMA, Adrogué, B1846DSK Buenos Aires, Argentina; ^3^LSU School of Medicine, Shreveport, LA 71103, USA

The authors' names were incorrectly listed as Docampo Jorge, Gonzalez Nadia, Vazquez Claudio, Morales Carlos, and Gonzalez-Toledo Eduardo; this error is corrected here.

